# The baseline bubble inclinometer measurement of sagittal thoracic spinal range of motion is reliable: Validated by optoelectronic motion capture system

**DOI:** 10.1177/10538127251357101

**Published:** 2025-07-22

**Authors:** Ziang Jiang, Jiling Ye, Rongshan Cheng, Qiang Zhang, Lili Xu, Tsung-Yuan Tsai

**Affiliations:** 1 Med-X Research Institute, School of Biomedical Engineering, Shanghai Jiao Tong University, Shanghai, China; 2 Department of Health Sciences and Technology, Institute for Biomechanics, ETH Zürich, Zurich, Switzerland; 3 Rehabilitation department, Shanghai Ninth People's Hospital, Shanghai Jiao Tong University School of Medicine, Shanghai, China

**Keywords:** thoracic vertebrae, range of motion, motion capture, baseline bubble inclinometer, validation study

## Abstract

**Background:**

The thoracic spinal range of motion (ROM) is a commonly used in pathological and functional assessment. Baseline bubble inclinometers are one of the most frequently employed thoracic ROM measurement methods. However, there is currently no consensus on the accuracy and standardized procedure of their utilization.

**Objective:**

The purpose of this study is to validate the accuracy of baseline bubble inclinometers in measuring the sagittal thoracic spinal ROM and to propose the standard guideline for their utilization.

**Method:**

28 participants were recruited for this study. The maximum thoracic spinal ROM during flexion and extension was measured using inclinometers, with the optoelectronic motion capture system (Vicon) serving as the control group.

**Result:** The thoracic spinal ROM during flexion was 14.5 ± 10.5°; during extension was 19.0 ± 9.2°, and the total ROM was 33.5 ± 14.0°. The inclinometers showed moderate to high correlations with the Vicon results, particularly in measuring flexion ROM, which exhibited the highest effectiveness (r = 0.84∼0.89). The accuracy of the inclinometers was enhanced by ensuring a cervical nodding and fixation position. Additionally, it was observed that females were more suitable candidates for thoracic spinal ROM measurement using inclinometers, as they exhibited higher correlations with the Vicon results.

**Conclusion:**

This study successfully validated the accuracy of the inclinometer as a convenient thoracic spinal ROM measurement method, that can save significant time for physiotherapists in clinical settings. The measurements obtained in this study may serve as a preliminary reference for the thoracic spinal ROM in healthy individuals and standardized protocols for using the baseline bubble inclinometer.

## Introduction

The thoracic spinal range of motion (ROM) refers to the maximum angle between the vertebral bodies from T1 to T12 during movements.^1^ It is a crucial indicator for evaluating the spinal function and vertebral angular alignments during flexion and extension.^1,2^ Abnormalities or limitations in the sagittal ROM of the thoracic spine can arise from factors such as degenerative changes, vertebral malformations, and spinal postoperative outcomes.^3^ Therefore, having a precise and convenient method to measure the sagittal thoracic spinal ROM is of significant importance for diagnosing musculoskeletal disorders and evaluating postoperative outcomes.

The measurement of sagittal ROM in the thoracic spine is a complex task due to the synergistic interactions of multiple segments involved in thoracic spinal movement. Currently, there are various methods and techniques available for clinical measurement of thoracic spinal mobility and flexibility, including radiographs,^4–6^ 3D ultrasound,^6,7^ baseline bubble inclinometer,^5,6,8^ flexicurve,^5,6^ inertial measurement unit (IMU),^9^ electrogoniometry,^10^ or based on cadaver specimens.^11^ While fluoroscopic techniques such as MRI and X-rays have been considered the most precise methods for measuring intervertebral patterns and the range of motion of vertebral segments,^12,13^ their application is limited due to radiation exposure, operational complexity, and time-consuming procedures. In clinical settings, there is a preference for noninvasive and convenient methods that offer high accuracy in measuring the thoracic spinal ROM for patients.

Previous studies have demonstrated the reliability of motion capture and analysis techniques, with high correlations and moderate to good intraclass correlation coefficients (ICCs) between radiographic and optical marker measurements.^3,5^ During the measurement process, the optical motion capture system has shown impressive accuracy, marker positioning frequently can achieve up to 0.1 mm.^14^ It has proven to be reliable in tracking vertebral motion during flexion and extension and showed efficacy in the analysis of the functional performance of spinal alignment disorders such as scoliosis, thoracolumbar kyphosis, etc..^15,16^ However, the preparation process for optical motion capture can be time-consuming. This includes calibrating the infrared field to determine the external parameters between the capture cameras, as well as the palpation and confirmation of the skin landmarks placement. Failure to properly carry out these steps can lead to errors in spinal kinematic data recording, as the system is susceptible to random errors and soft tissue artifacts.^3^ Although numerous studies used the optical motion capture technique to observe the spinal sagittal range of motion, there is still a clinical requirement for a more convenient and reliable maneuver.^5,15–17^

The baseline bubble inclinometer has been widely used as a cost-effective clinical solution for measuring the spinal sagittal ROM.^8,18–20^ The ROM was calculated by comparing the segmental kyphotic angles in the maximum flexion and extension.^8^ While inclinometers have found popularity in orthopedics and rehabilitation for measuring spinal ROM,^8,19,20^ the accuracy of this method has not been quantitatively validated. Additionally, the placement of the inclinometer typically crossed the C7 and T1 bony landmarks at the start point of the thoracic spine and the T12 and L1 at the end.^8,19^ This means that movement of the head and neck can affect the measurement results. However, it has not been clearly defined whether the cervical vertebra should be fixed in a neutral position during measurement, and the impact of the head-neck has not yet been quantified. Currently, there is no standardized utilization specification to ensure consistent measurement and prevent potential systematic errors that may occur during the measurement process. Additionally, there is a known difference in spinal flexibility between males and females,^21^ which may affect the applicability of the baseline bubble inclinometers.

The aim of this study was: (1) To evaluate the accuracy of the baseline bubble inclinometer in measuring thoracic spinal ROM, using an optoelectronic motion capture system as the reference standard. (2) To propose a standardized protocol for inclinometer application and to investigate the influence of gender on measurement accuracy. (3) To provide additional reference data on thoracic spinal ROM in healthy individuals.

## Method

### Subjects demographics

The Internal Review Board approval was granted by the institutional ethics committee in Shanghai Ninth People's Hospital (SH9H-2021-TK12-1). At least 20 participants were planned to be recruited in this study as the suggested adequate number of subjects required is 19 to reach a significance level of 5% and a power of 80.^22,23^ The inclusion criteria were as follows: (1) Healthy participants with no history of surgery or trauma affecting the cervical, thoracic, or lumbar spine that could impact the experiment; (2) Individuals with full capacity to independently perform flexion and extension of the lumbar and thoracic spine; (3) No obvious kyphosis or scoliosis (Cobb < 10°); and (4) body mass index (BMI) ≤ 30. Participants who did not meet these criteria were excluded from the experiment.

### Data collection tools

#### Baseline bubble inclinometer measurement

A physical therapist with over five years of clinical experience placed two bubble inclinometers (Theratools, Zhengzhou, China) on the spinous process of T1 and T12 and adjust them to zero. Thoracic sagittal ROM was calculated by the summation of the inclinometer placed over the spinous process, including spinal extension and flexion.

**Figure 1.**
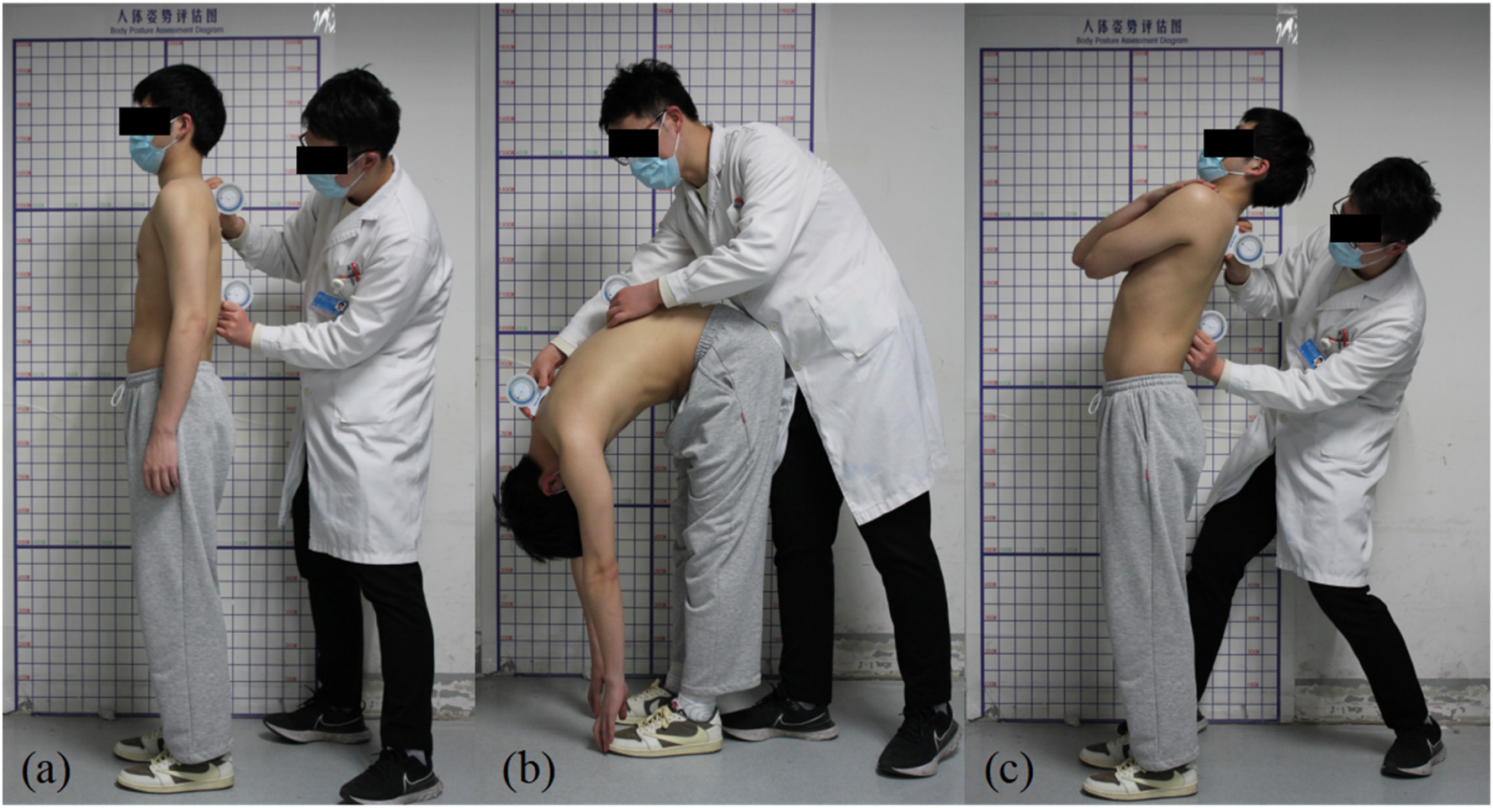
Measurement process of thoracic ROM. (a) Start position; (b) Maximal flexion; (c) Maximal extension.

Participants are required to complete the following actions (Figure 1):

1. Start position (standing upright in a relaxed position, focusing on a marker at eye level, feet shoulder width apart, knees straight, arms hanging by the side)
2. Maximal flexion (keep legs straight, trunk flexed as far as comfortably possible in an attempt to curl the head into the knees)
3. Maximal extension (keep legs straight, put hands around the chest, head in a neutral position, trunk extended comfortably as far as possible)

The actions were performed in cervical nodding and fixation situation (CNF) and natural conditions. Under the CNF condition, participants were instructed to maintain the cervical spine in an upright and stabilized position, similar to a nodding posture, throughout all measurement procedures. Conversely, in the natural condition, cervical posture was self-regulated based on the participant's habitual alignment.

#### Optoelectronic motion capture measurement

**Figure 2.**
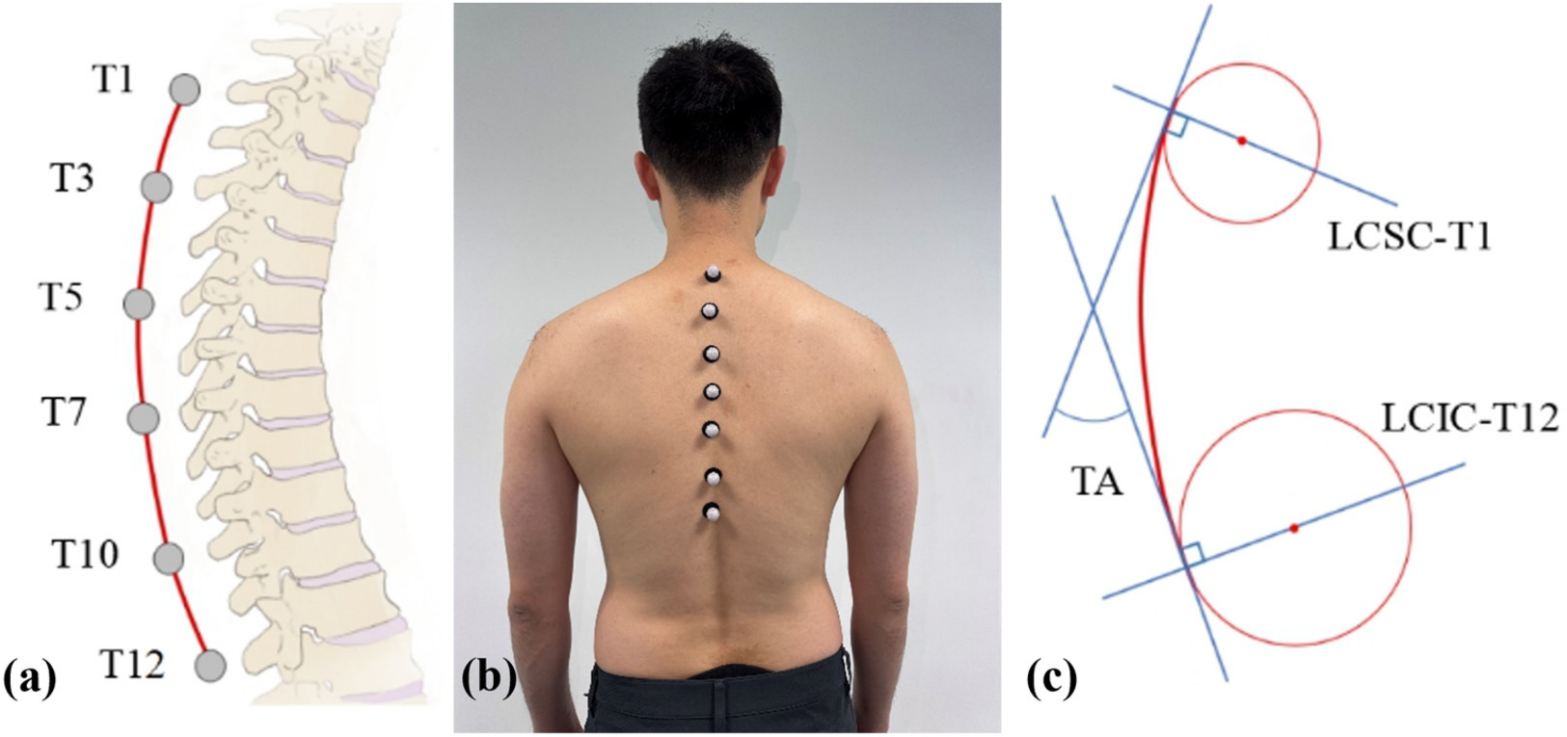
Optical motion capture process and the ROM calculation illustration. (a) passive infrared reflective markers placement; (b) Illustration of the attachment of infrared reflective marker sets; (c) Thoracic angle definition.

The back of each participant was appropriately exposed to manually find the spinous process location of the T1, T3, T5, T7, T10, and T12 vertebrae. A series of 14 mm infrared reflective markers were secured on these positions accordingly as Figure 2(a) and (b). All positions of the marker sets were confirmed through the skin above the vertebrae by a professional physiotherapist (J.Y.). Through this placement strategy, all the thoracic vertebrae can be covered and described. The placement of markers was chosen with thinner soft tissue thickness to decrease the soft tissue artifacts during movement under different postures,^24^ which is referred to as the method proposed by Kim et al. and Ekizos et al.^25,26^ with certain modifications.

The test postures were performed the same as the inclinometer measurement procedure, that the participants were instructed to repeat the actions during measurements. Each subject kept the trained posture for at least 10 s to reach a stable static record for analysis by 6 Vero v2.2 Vicon camera motion capture system (Vicon motion systems LTD unit 6, Vero v2.2, Oxford, United Kingdom). The data collection frequency was set to 200 Hz, with the motion capture environment optimized to minimize interference and eliminate obstructions. The consistent and accurate tracking of all infrared reflective markers was ensured throughout the data collection process. The recording phase was secretly processed to avoid postural deviation caused by emotional factors. The recorded data was imported into Vicon Nexus software (Vicon, Oxford, UK), and the average 3D position of the reflective markers was processed by the Matlab (MATLAB; The MathWorks, Natick, MA) program to calculate the thoracic angle (TA) during flexion or extension.

The calculation of TA is shown in Figure 2(c). A continuous curve that can describe the thoracic flexion degree is fitted by cubic spline interpolation according to the position of the markers. To precisely describe the inclination of the T1 and T12 vertebrae， two circles were fitted from the superior and inferior margins of the curve, respectively. TA was represented as the angle between the line connecting the center of the superior circle and T1 (LCSC-T1) and the line connecting the center of the inferior circle and T12 (LCIC-T12). Geometrically, the TA is equal to the angle between the tangent lines of LCSC-T1 and LCIC-T12 shown in Figure 2(c). The difference between the flexion and extension TA with the upright TA respectively represents the ROM of thoracic flexion and extension. The total ROM is defined as the summation of both the flexion and extension ROM. The TA calculation method has already been validated in previous studies.^16,17,27^ Notably, after completing the measurements under the baseline bubble inclinometer condition, each participant was given a 10–15 min rest period before proceeding to the Vicon system measurement.

### Statistical analysis

SPSS version 26.0 (Chicago, IL, USA) was used for descriptive statistics. All values including flexion, extension, and total ROM are expressed as the mean ± standard deviation. The TA during flexion and extension measured from Vicon system and inclinometer was also presented descriptively. Participants of different genders were analyzed separately to identify any gender similarities and differences in variables.

The Shapiro–Wilk test was employed to assess the normality of the data distribution, in order to determine the appropriate statistical tests for subsequent analysis. Pearson correlation coefficients (r) were used to evaluate the correlations between the thoracic spinal ROM in the sagittal plane measured from both the inclinometer and Vicon system. Student's T-test was used to evaluate the significance of the difference between the results measured through the two different methods. All tests were two-tailed, and statistical significance was defined as p < 0.05.

## Result

A total of 28 participants (13 male, and 15 female) were recruited for this investigation. Their combined mean age was 22.9 ± 4.5 years, mean height 170.1 ± 8.2 cm, mean weight 68.3 ± 18.3 kg, and mean BMI 23.1 ± 4.6 kg/m^2^. In flexion, the ROM measured through Vicon system in the CNF group was 13.0 ± 10.4°, while the result of the inclinometer was 14.5 ± 10.5°. The measurement showed a high correlation that *r* performed 0.86, and no significant difference was shown (*p* = 0.581), while the mean difference is −1.6°. When the cervical is not fixed, the correlation performed *r* = 0.75, and also no significant difference was showed (*p* = 0.126), but the mean difference reached −4.3. The difference of both Vicon system and inclinometer measured in CNF position and natural position situations showed significance respectively (p = 0.003; 0.000). For males, the CNF group showed a high correlation with the Vicon system result and performed no significant difference (*r* = 0.84, *p* = 0.541). And in the natural position group, a moderate correlation was shown (*r* = 0.677), while the difference is not significant (*p* = 0.174). For females, the CNF group showed a high correlation as *r* = 0.89 and no significant difference as *p* = 0.856. And natural position group showed a high correlation as r = 0.81 and no significant difference as *p* = 0.401. The difference between Vicon system and inclinometer in both genders was significant. (Table 1)

**Table 1.** The measurement of thoracic spinal ROM in flexion.

	Flexion ROM (CNF)					Flexion ROM (Natural)						
	Vicon^#*^	inclinometer^#*^	Difference*	r	p	Vicon^#*^	inclinometer^#*^	Difference*	r	p	Pv	Pi
All	13.0 ± 10.4	14.5 ± 10.5	−1.6	0.86	0.581	21.7 ± 9.9	26.0 ± 10.5	−4.3	0.75	0.126	0.003	0.000
Male	13.4 ± 9.3	16.0 ± 10.7	−2.5	0.84	0.541	21.1 ± 8.1	26.3 ± 10	−5.2	0.68	0.174	0.042	0.023
Female	12.5 ± 11.3	13.3 ± 10.1	−0.7	0.89	0.856	22.2 ± 11.3	25.7 ± 10.9	−3.6	0.81	0.401	0.032	0.004

# Expressed as mean ± standard deviation; * Expressed in °.

r: Pearson correlation coefficient; p: student's t-test p-value indicating the significance of differences between Vicon system and inclinometer groups.

Pv: student's t-test p value between measurement of Vicon system when participants are in CNF and natural conditions.

Pi: student's t-test p value between measurement of inclinometer when participants are in CNF and natural conditions.

In extension, the ROM measured through Vicon system in the CNF position was 14.9 ± 8.5°, while the result of the inclinometer was 19.0 ± 9.2°. The measurement showed a moderate correlation that *r* performed 0.62, and no significant difference was shown (*p* = 0.098). When in the natural position group, the correlation is also moderate which performed r = 0.5 but showed a significant difference (*p* = 0.000). The difference in Vicon system measurement in CNF and natural position groups showed no significance as *p* = 0.642, while the inclinometer result showed *p* = 0.004. For males, both CNF and natural groups showed moderate correlation as *r* = 0.33 and 0.32 respectively. No significant difference was shown in the CNF group as *p* = 0.315 and a significant difference was shown in the natural position group as *p* = 0.001. For females, the CNF group also showed a moderate correlation as *r* = 0.72 and no significant difference as *p* = 0.178. And natural position group showed a high correlation as *r* = 0.60 but showed a significant difference (*p* = 0.004). The difference in inclinometer in both genders was significant. In the CNF group, the Vicon system result showed an insignificant difference of *p* = 0.815, but the natural position group showed a significant difference (*p* = 0.001) (Table 2).

**Table 2.** The measurement of thoracic spinal ROM in extension.

	Extension ROM (CNF)					Extension ROM (Natural)						
	Vicon^#*^	inclinometer^#*^	Difference*	r	p	Vicon^#*^	inclinometer^#*^	Difference*	r	p	Pv	Pi
All	14.9 ± 8.5	19.0 ± 9.2	−4.1	0.62	0.098	13.8 ± 10.0	26.14 ± 8.5	−12.4	0.50	0.000	0.642	0.004
Male	12.9 ± 7.4	15.8 ± 6.7	−3.0	0.33	0.315	13.7 ± 9.4	26.2 ± 6.2	−12.4	0.32	0.001	0.815	0.001
Female	16.7 ± 8.9	21.7 ± 8.9	−5.0	0.72	0.178	13.8 ± 10.6	26.1 ± 10.1	−12.3	0.60	0.004	0.438	0.262

# Expressed as mean ± standard deviation; * Expressed in °.

r: Pearson correlation coefficient; p: student's t-test p-value indicating the significance of differences between Vicon system and inclinometer groups.

Pv: student's t-test p value between measurement of Vicon system when participants are in CNF and natural conditions.

Pi: student's t-test p value between measurement of inclinometer when participants are in CNF and natural conditions.

The total ROM range recorded by Vicon system showed as 27.9 ± 10.8°, and the inclinometer was 33.5 ± 14.0°, with a mean difference of −5.6°, in the CNF group. And moderate correlation and no significant difference were shown (*r* = 0.68; *p* = 0.103). The Vicon system result recorded in the natural position group was significantly different from the inclinometer result as p was shown as 0.000, and a moderate correlation was shown (*r* = 0.55). The difference between both Vicon system and inclinometer methods between CNF and natural position groups was significant (*Pv* = 0.010; *Pi* = 0.000). For males, a moderate correlation was shown in the CNF group and showed no significant difference (*r* = 0.34; *p* = 0.103). And in the natural position group, the low correlation was shown with a significant difference (*r* = 0.09; *p* = 0.000). For females, a moderate correlation was also shown in the CNF group and showed no significant difference (*r* = 0.79; *p* = 0.303). And in the natural position group, a moderate correlation was shown but with a significant difference (*r* = 0.69; *p* = 0.007). (Table 3)

**Table 3.** The measurement of total thoracic spinal ROM.

	Total ROM (CNF)					Total ROM (Natural)						
	Vicon^#*^	inclinometer^#*^	Difference*	r	p	Vicon^#*^	inclinometer^#*^	Difference*	r	p	Pv	Pi
All	27.9 ± 10.8	33.5 ± 14.0	−5.6	0.68	0.103	35.4 ± 9.9	52.1 ± 13.8	−16.7	0.55	0.000	0.010	0.000
Male	26.3 ± 7.2	31.8 ± 11.1	−5.1	0.34	0.161	34.8 ± 5.6	52.5 ± 9.7	−17.6	0.09	0.000	0.004	0.000
Female	29.2 ± 13.0	35.0 ± 15.9	−5.8	0.79	0.303	36.0 ± 11.8	51.9 ± 16.5	−15.9	0.67	0.007	0.171	0.010

# Expressed as mean ± standard deviation; * Expressed in °.

r: Pearson correlation coefficient; p: student's t-test p-value indicating the significance of differences between Vicon system and inclinometer groups.

Pv: student's t-test p value between measurement of Vicon system when participants are in CNF and natural conditions.

Pi: student's t-test p value between measurement of inclinometer when participants are in CNF and natural conditions.

## Discussion

In this study, we discovered that the baseline bubble inclinometer can achieve a high level of accuracy with moderate to high correlation between the inclinometer measurements and the results obtained from the Vicon system, and there were no significant differences between the two methods. The inclinometer was particularly effective in measuring spinal flexion. Females tend to have a higher range of motion in thoracic spinal compared to males. The accuracy of the inclinometer measurements was found to be higher in females compared to males. Fixing the cervical vertebra in a neutral position during the measurement process is crucial for improving the accuracy of the inclinometer.

The total ROM of the thoracic spine is 33.5 ± 14.0° measured from inclinometers in this study is similar to the result of Morita *et al*. who reported a ROM of 31.7 ± 11.3° in 50 healthy adults measured using computed tomography (CT) scanning.^1^ Ignasiak et al. utilized the Vicon system to measure thoracic flexion and found a range of ROM of 48.9° ± 8.8°.^27^ The results of previous studies showed consistency with the measurement results obtained from this study, which to some extent proves the accuracy of the measurement process.

The CNF group demonstrated superior performance in inclinometer-based measurements of thoracic spinal ROM compared to the natural posture group. Across all ROM types and both genders, the CNF group showed higher correlation coefficients between inclinometer and Vicon system results. Importantly, all statistically significant differences (p < 0.05) between the two measurement methods occurred in the natural posture group, whereas no significant differences were observed in the CNF group. This suggests that the cervical instability may introduce variability that compromises the accuracy of inclinometer measurements. Additionally, except for extension ROM in female group, significant differences (Pi < 0.05) were found between inclinometer measurements in CNF and natural conditions across most subgroups. The Vicon system results also reflected significant differences between these two postures in several cases, including flexion and total ROM among male participants and the overall sample. These findings underscore the importance of stabilizing the cervical spine during measurement. Maintaining a neutral, fixed cervical posture is essential to minimize soft tissue artifacts and ensure the reliability and consistency of inclinometer-based thoracic ROM assessments.

In comparison, inclinometer measurements demonstrate the highest accuracy in assessing flexion movement, as indicated by the highest calculated r-values in flexion action. This could be attributed to the presence of fewer soft tissue artifacts during flexion, as the skin coverage is thinner.^28,29^ Moreover, it is possible that controlling the participants’ heads during extension was more challenging. As a result, the inclinometers can be considered one of the best options for evaluating the flexion function in patients. However, the application of inclinometers can also be expanded to include measurements of flexion, extension, and the total ROM, as none of the p-values in both gender group and the group with all subjects showed significant difference when the cervical vertebra is fixed in nodding position for these three kinds of ROM in the thoracic spine. Moreover, in the CNF situation, all correlations exhibited R-values ranging from moderate to high, further supporting the reliability of inclinometer measurements.

In addition to maintaining a fixed position of the cervical vertebra in a nodding position, which ensures a more standardized measurement process, the gender also plays a significant role in the accuracy of inclinometer ROM measurements. In both flexion and extension, and the total ROM calculations, the female groups consistently demonstrated higher correlations compared to the male groups, and the male groups all performed the lowest correlations. This can be attributed to the fact that males typically have higher muscle mass,^30^ and the muscle function in the back region may introduce additional soft tissue artifacts.^31^ Consequently, the application of inclinometers in females is likely to yield better effectiveness and accuracy.

This research, inevitably has some limitations that should be acknowledged. Firstly, there may be an uncontrollable offset in the position of infrared reflective markers fixed on the skin in the frontal or transverse plane in spinal motion. To mitigate this potential deviation, we projected the markers uniformly on the sagittal plane in the Vicon system data, a method in the previous study.^16^ Secondly, the majority of participants recruited in the present study fell within or even above the normal BMI range. Therefore, further validation is needed to confirm the reliability of inclinometer measurements across different BMI ranges and other individual characteristics, particularly in more diverse populations.^29^ Thirdly, it is important to acknowledge that the sample size in this study is relatively small. Although the sample size was limited, the study successfully identified significant differences and demonstrated moderate to high statistical correlations. Furthermore, the sample size in this study exceeds that of many similar studies.^7,15–17,26–28^ The range of measurement results observed also corresponds closely with findings from previous research,^1,27^ further validating the reliability of the sample size in this experiment. Lastly, To our knowledge, there is currently no established gold standard for measuring thoracic spine ROM. The comparison with motion capture methods was conducted as one of the relatively accurate approaches currently available. However, the results presented in this descriptive study suggest that the baseline bubble inclinometer can achieve relatively accurate measurements to be a kind of clinical reference.

## Conclusion

This study validates the accuracy of the Baseline Bubble Inclinometer for measuring thoracic spinal range of motion (ROM) and introduces a standardized measurement protocol to improve consistency and reliability. The findings highlight the importance of maintaining a fixed cervical posture—achieved through cervical nodding—for ensuring measurement accuracy. The inclinometer demonstrated strong versatility and effectiveness in assessing flexion ROM across both genders. However, for extension ROM and total ROM calculations, the device showed greater accuracy in female participants. While it remains reasonably accurate in males, alternative methods may enhance measurement precision in this population. In addition, this study offers reference data on thoracic flexion, extension, and total ROM in healthy individuals, contributing to the current understanding of normative spinal mobility. These results hold potential clinical value in fields such as physical rehabilitation and spinal surgery.
